# Gradient fluid shear stress regulates migration of osteoclast precursors

**DOI:** 10.1080/19336918.2019.1619433

**Published:** 2019-05-25

**Authors:** Yan Gao, Taiyang Li, Qing Sun, Bo Huo

**Affiliations:** Biomechanics Lab, Department of Mechanics, School of Aerospace Engineering, Beijing Institute of Technology, Beijing, P. R. China;

**Keywords:** Parallel-plate flow chamber, bone remodeling, cell mechanics, cell migration, calcium signaling pathway

## Abstract

Cell migration is highly sensitive to fluid shear stress (FSS) in blood flow or interstitial fluid flow. However, whether the FSS gradient can regulate the migration of cells remains unclear. In this work, we constructed a parallel-plate flow chamber with different FSS gradients and verified the gradient flow field by particle image velocimetry measurements and finite element analyses. We then investigated the effect of FSS magnitudes and gradients on the migration of osteoclast precursor RAW264.7 cells. Results showed that the cells sensed the FSS gradient and migrated toward the low-FSS region. This FSS gradient-induced migration tended to occur in low-FSS magnitudes and high gradients, e.g., the migration angle relative to flow direction was approximately 90° for 0.1 Pa FSS and 0.2 Pa mm^−1^ FSS gradient. When chemically inhibiting the calcium signaling pathways of the mechanosensitive cation channel, endoplasmic reticulum, phospholipase C, and extracellular calcium, the cell migration toward the low-FSS region was significantly reduced. This study may provide insights into the mechanism of the recruitment of osteoclast precursors at the site of bone resorption and of mechanical stimulation-induced bone remodeling.

## Introduction

In vivo migration of cells generally occurs under fluid flow in blood vessels or interstitial cavities through metastasis of cancer cells,[1] immune response [2], embryonic development [3], and tissue regeneration [4]. A large number of in vitro studies basing on microfluidic technology or parallel-plate flow chamber were performed to investigate the effect of fluid shear stress (FSS) on cell migration. For example, flow stimulation with different chemical concentration gradients showed obvious effect on the migration of nerve cells, endothelial cells, or cancer cells by using microfluidic devices [5,6]. Another study showed that when FSS was exerted on endothelial cells cultured on nanofiber membranes, the cells tended to migrate along the well-aligned nanofiber direction to maintain the integrity of intercellular connections [7]. In some cases, however, cells may display unexpected behavior under flow stimulation. For example, MDA-MB-231 cancer cells are in high density or inhibit the CCR-7 receptor migrate against the flow direction [8]. The present study reported another unexpected migration of osteoclast precursors under gradient FSS.

The migration of mononuclear osteoclast precursors starts from the capillaries of bone microcirculation system and ends at the periosteum close to bone surfaces that encompass the trabecular and intramedullary cavities. The deformation of the bone mineral matrix caused by mechanical loading can drive fluid flow within cavities, thereby inducing FSS on the bone surface and adherent bone cells [9–11]. When osteoclasts enter the bone surface, they will experience the shear stress from interstitial fluid flow, similar to osteoblasts. Given that the FSS level inside a microdamage should be less than that on the bone surface, osteoclast precursors may migrate toward the low-FSS regions, such as around large-scale microcrack, explaining the phenomenon of targeted bone remodeling [12–14]. However, this speculation lacks evidence from experimental observations.

Our previous study found that osteoclast precursors migrated along the flow direction in parallel-plate flow chamber, and the migration speed is proportional to the FSS magnitude, which is regulated by calcium signaling pathway [15]. However, when we used a novel cone-plate flow chamber to apply gradient FSS on osteoclast precursors, the cells did not migrate along the flow direction but toward the region with low wall FSS (not published). The cone-plate flow chamber may be involved with secondary fluid flow. Although the migration angle of the cells toward the low-FSS region is larger than the angle of secondary flow, further evidence must be obtained to reconfirm this novel phenomenon.

This study established the FSS gradient by using a modified parallel-plate flow chamber. Osteoclast precursor RAW264.7 cells were exposed to FSS with different magnitudes and gradients, and the parameters of cell migration were analyzed. Furthermore, the effect of calcium signaling pathways of mechanosensitive cation channel (MSCC), endoplasmic reticulum (ER), phospholipase C (PLC), and extracellular calcium on flow-induced cell migration was studied.

## Materials and methods

### Gradient parallel-plate flow chamber

**Figure 1.**
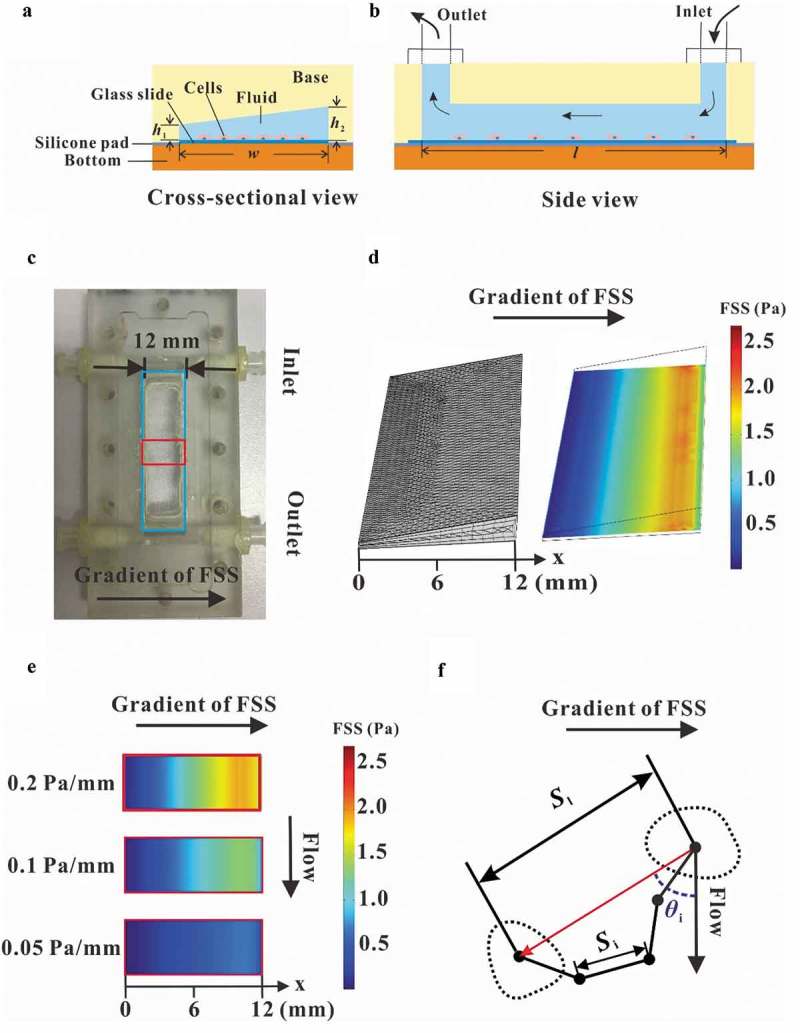
Establishment of gradient parallel-plate flow chamber. Schematic diagrams of gradient parallel-plate flow chamber along the cross-sectional view (a) and side view (b). (c) Photo of the base plate of the custom-made gradient parallel-plate flow chamber, wherein the arrow represents the direction of the FSS gradient. (d) FE mesh and wall FSS of gradient parallel-plate flow chamber indicated by the blue box in (c). (e) Numerical simulation results of wall FSS on the bottom surface at the location indicated by the red box in (c). The x-axis is defined along the FSS gradient. (f) Definition of migration parameters. The black dots indicate the centroids of a migrating cell at different times. *S*_i_ is the distance of cell movement in a given time interval, *S*_l_ is the distance of a cell away from its initial position in a given time, and *θ*_i_ is the angle between the flow and migration directions of one cell relative to its initial position.

We designed and fabricated a modified parallel-plate flow chamber to establish flow field with different gradients of wall FSS. The difference of this device compared with a traditional parallel-plate flow chamber is the leaning top cover so that it is not parallel to the bottom plate along the direction perpendicular to the longitudinal axis of the chamber (Figure 1(a)). Finally, the dimension of the flow channel is of 12 mm wide (*w*), 50 mm long (*l*), and 0.2 mm high (*h*_1_) at one side but 0.8 mm high (*h*_2_) at the other side. Wall FSS exerted on cells would be controlled by specifying the rotation speed of the peristaltic pump. Therefore, this so-called gradient parallel-plate device was able to provide a microenvironment of gradient FSS for the cells.

## Numerical simulation

Finite element analysis (FEA) method was used for numerical simulation on flow field in the flow chamber. An FEA model was constructed considering the geometric parameters of parallel-plate flow chamber. The inlet pressure of the chamber was assigned as 300 Pa, and the outlet pressures were 0, 100, and 200 Pa to form three FSS gradients of 0.2, 0.1, and 0.05 Pa mm^−1^, respectively. The above pressure differences are similar to those in lacunar-canalicular system within the bone [16]. COMSOL Multiphysics software was adopted for FEA.

## Particle image velocimetry (PIV) experiment

PIV technique was adopted to indicate the direction of fluid flow in the gradient parallel-plate flow chamber. Briefly, fluorescent beads of carboxylate-modified polystyrene with mean diameter of 0.5 μm (Sigma, USA) were suspended in deionized water (1:5) with a vortex mixer to ensure uniform distribution. After the parallel-plate flow chamber ran stably for 5 min under fluorescence microscope, the movement of beads was recorded at 10 fps with a camera, and the focal plane was 0.1 mm above the plate, shown as a straight-like trajectory in the images. The movement parameters of the beads, including speed and orientation, were then analyzed by an image-processing program based on MATLAB software. The linear velocity *v*_p_ was obtained by dividing the length *L*_s_ of a trajectory arc with the exposure time of 5 ms for one fluorescent image. Wall FSS $\tau_{w}=\eta \cdot v_{p}/h_{p}$, in which the distance *h*_p_ is 0.1 mm, and the viscosity *ƞ* is 1 × 10^−1^ Pa s. This wall FSS value was compared with that from numerical simulation. Digital image analysis was performed with Image J and MATLAB software.

## Cell culture

The RAW264.7 cell line was derived from a tumor developing in a BAB/14 mouse, inoculated with Abelson murine leukemia virus [17]. This cell line has been employed for studying the differentiation and activity of osteoclast due to its expression of Receptor activator of NF-κB (RANK) [18,19]. In this study, osteoclast precursor RAW264.7 cells (European Collection of Cell Cultures, UK) were cultured in Dulbecco’s modified Eagle’s medium (DMEM, Hyclone, USA) supplemented with 10% fetal bovine serum (Gibco, USA), 100 unit mL^−1^ of penicillin (Sigma, USA), and 100 unit mL^−1^ of streptomycin (Sigma, USA) at 37°C and 5% CO_2_.

## Time-lapsed imaging for cell migration and inhibition of calcium signaling pathways

RAW264.7 cells were cultured on Permanox® cell culture slide (NUNC, USA) for 24 h before being exposed to FSS stimulation for allowing their adhesion on the substrate. The surface of the culture slide was pretreated with Type I collagen and was suitable for cell growth [20]. Then, the slide was mounted with flow chamber. After the chamber was connected with a peristaltic pump, live cell imaging was recorded for 2 h at 30 min intervals. Migration trajectory of each cell was observed, and the migration parameters of distance, speed, and orientation were analyzed. To study the effect of calcium signaling pathways on the migration of RAW264.7 cells under gradient FSS, four pharmacological agents were employed. Similar to our previous studies [15,21], the agents were incubated with cells before being exposed to FSS. In brief, 10 µM gadolinium chloride (Gd, Sigma, USA) was supplied as the MSCC blocker. The calcium stored in the ER was depleted by 1 µM thapsigargin (TG, J&K Chemicals, China). U-73,122 (Darmstadt, Germany) of 10 µM was adopted to inhibit PLC. For the above blocking tests, 10 min incubation of chemical reagents was applied. Calcium-free DMEM medium (Gibco, USA) was used to remove the extracellular calcium.

## Statistical analysis

Data were presented as mean±standard deviation (SD). Statistical analysis was performed using a one-way analysis of variance (ANOVA) with Tukey’s post hoc test for multiple comparisons to determine the statistical differences between the mean values of different groups. Data were presented as mean±SD if not specifically claimed. Each group in this study was repeated at least thrice. p < 0.05 was considered statistically significant.

## Results

### Parallel-plate flow chamber provides the flow field with gradient FSS

**Figure 2.**
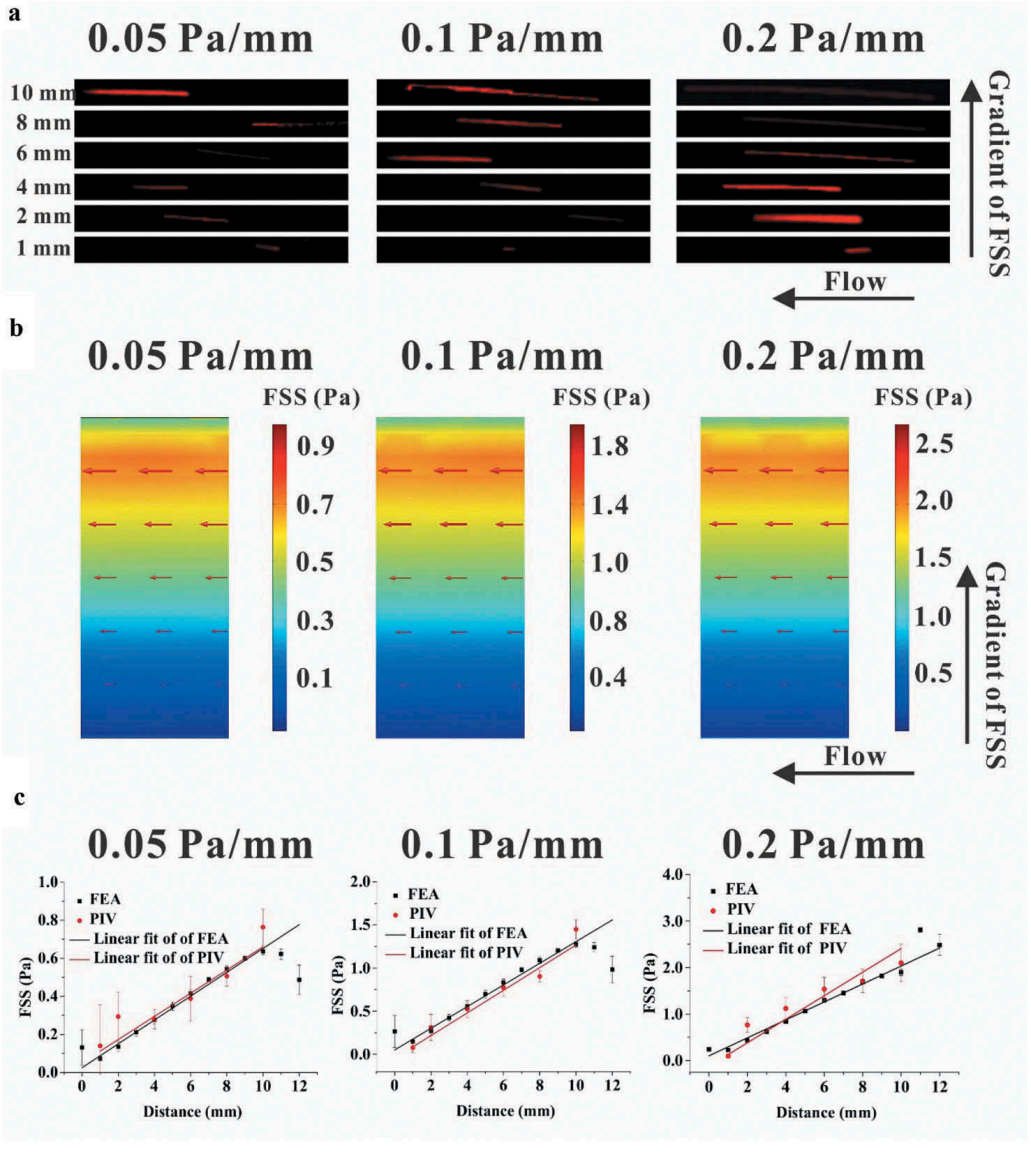
Comparison between the results of PIV measurement and FEA simulation. (a) Traces of fluorescent latex beads recorded at different locations for different gradient FSS fields. (b) Wall FSS in the three FEA models with different FSS gradients, wherein the red arrow represents the direction and magnitude of flow velocity. (c) Statistical analysis of wall FSS at different distances calculated based on the results from PIV measurement and FEA simulation. The regression coefficient for numerical simulation and PIV measurement are 0.9331 and 0.9507 for 0.05 Pa mm^−1^, 0.9345 and 0.9811 for 0.1 Pa mm^−1^, and 0.9793 and 0.9667 for 0.2 Pa mm^−1^, respectively.

A modified parallel-plate flow chamber was designed in this study to establish a gradient FSS field on the base plate (Figure 1(a,b)), in which the wedges on the transparent cover plate constructed the flow channel with a trapezoidal cross-section (Figure 1(c)). The mesh of parallel-plate flow chamber was locally refined to accurately simulate the wall FSS (Figure 1(d)). FEA results basing on this model obviously showed FSS gradient along the direction perpendicular to the flow direction, and changing the pressure difference produced the three FSS gradients, namely, 0.05, 0.1, and 0.2 Pa mm^−1^, respectively (Figure 1(e)). PIV technique was adopted to experimentally indicate the flow direction in the chamber. The images of fluorescent beads at different locations along the FSS gradient showed that the lengths of the particles’ trace seemed positively related to the locations (Figure 2a). FEA results revealed that the fluid velocity also increased along the FSS gradient (Figure 2(b)). In addition, flow direction was obviously along the chamber’s mainstream, and no secondary flow disturbance occurred. Statistical analysis further verified that the wall FSS based on PIV measurements or FEA simulations has linear relationship with the distance along the FSS gradient (Figure 2(c)). The above results indicated that we constructed a flow field with gradient FSS but without secondary flow.

## RAW264.7 cells migrate toward the low-FSS region that is dependent on FSS magnitude and gradient

**Figure 3.**
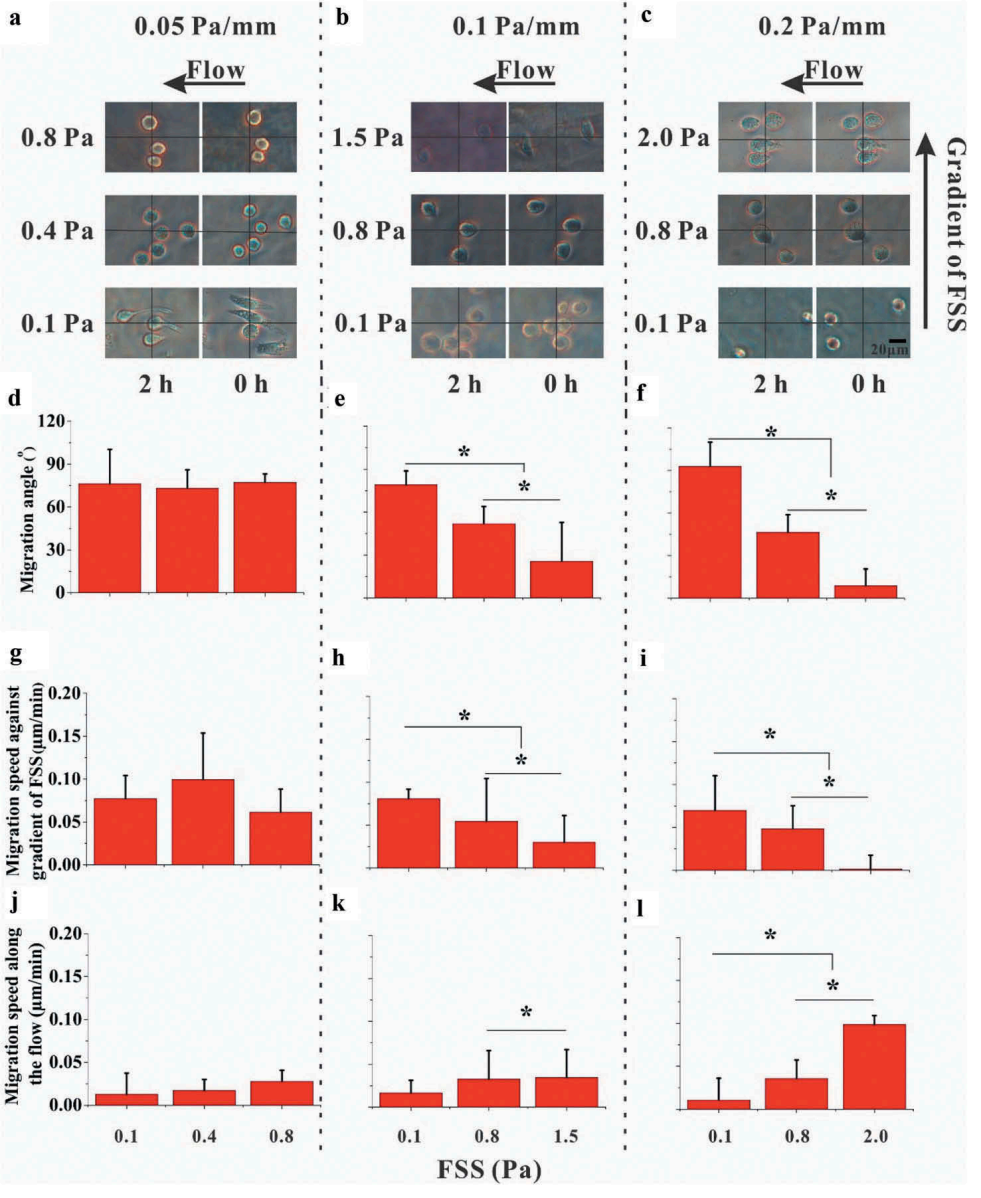
Effect of FSS magnitude on migration of RAW264.7 cells. (a, b, and c) Time-lapsed images of RAW264.7 cells with different magnitudes and gradients of FSS. (d, e, and f) Migration angle *θ*_i_ for three FSS gradients. (g, h, and i) Speed of cell migration along the gradient of FSS for three FSS gradients. (j, k, and l) Speed of cell migration along the flow direction for three FSS gradients. *, *p* < 0.05.

We found that most of cells disappeared from the field of view only after applying 2 Pa FSS for 2 h to 4 h (Supplementary Fig. S6A), suggesting that long-term and high-level FSS stimulation might cause the detachment or death of RAW264.7 cells. Therefore, we adopted 2 h as the stimulation time in the present study in order to compare the effect of different FSS magnitude on cell migration. RAW264.7 cells were exposed to fluid flow in the gradient parallel-plate flow chamber for 2 h, during which the images were captured at the locations with different magnitude of FSS (Figure 3(a, b, and c), Supplementary Movie S1). Then, the migration parameters were analyzed based on the images (Figure 1(f)). For the FSS gradient of 0.05 Pa mm^−1^, the average angle of cell migration relative to the flow direction after 2-h-FSS stimulation was approximately 75°, which was independent of the FSS magnitude (Figure 3(d)). In addition, no significant difference was observed in the migration speed along the flow direction or along the FSS gradient (Figure. 3(g, j)). However, for the large FSS gradient of 0.1 or 0.2 Pa mm^−1^, the angle of cell migration decreased with the increase of FSS magnitude (Figure 3(e, f)). Similarly, the migration speed along the FSS gradient did not reveal significant difference between different FSS magnitudes for 0.05 Pa mm^−1^ FSS gradient but was reduced by high FSS magnitude for 0.1 or 0.2 Pa mm^−1^ FSS gradient (Figure 3(g, h, and i)). The migration speed along the flow direction is contrary to the migration speed along the FSS gradient (Figure 3(j, k, and l)).

The relation between the directional migration of cells and the FSS gradient was further studied for two FSS levels of 0.1 and 0.8 Pa, which are in the physiological range of FSS in the postcapillary venules or osseous lacunae [11]. In 0.1 Pa FSS, the migration angle of cells for 0.2 Pa mm^−1^ was significantly greater than that for 0.05 or 0.1 Pa mm^−1^ (Supplementary Fig. S7A). However, the speed of cell migration along the flow direction or against gradient of FSS was independent of FSS gradient (Supplementary Figs. S7B and S7C). When the FSS magnitude was 0.8 Pa, the angle of cell migration for 0.2 or 0.1 Pa mm^−1^ FSS gradient was significantly less than that for 0.05 Pa mm^−1^ (Supplementary Fig. S7D). Similar trend was found for the migration speed against gradient of FSS but with no significant difference for the migration speed along the flow direction (Supplementary Figs. S7D and S7F). Therefore, the above results showed the migration of RAW264.7 cell toward the low-FSS region in a flow field with gradient FSS, especially when the FSS magnitude is relatively low.

## Migration of RAW264.7 cells toward the low-FSS region is reduced by inhibiting the calcium signaling pathways

**Figure 4.**
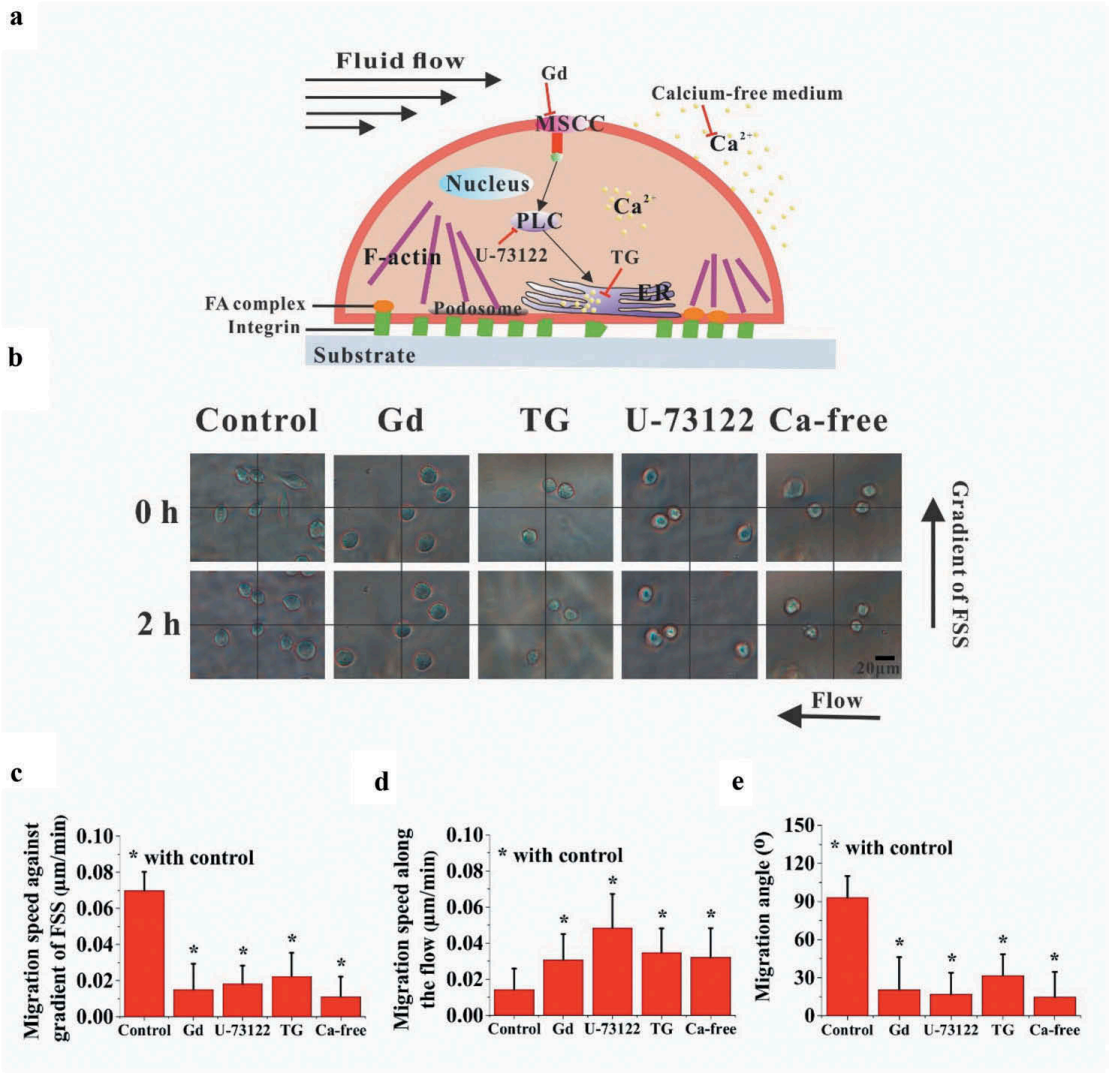
Effect of calcium signaling pathways on cell migration. (a) Schematic diagram of calcium signaling pathway and chemical treatments. (b) Bright-field images of RAW264.7 cells in the locations with large migration angle for 0.1 Pa mm^−1^ or 0.2 Pa mm^−1^. The effect of calcium signaling pathways on the migration speed along the FSS gradient (c) and along the flow direction (d). (e) Effect of calcium signaling pathways on the migration angle. *, *p* < 0.05.

To verify whether calcium signaling pathways may be involved in the above FSS gradient-induced cell migration, chemical treatments were adopted to inhibit the pathways of calcium transportation through MSCC and calcium release from ER (Figure 4(a)). The bright-field images of cells were captured in the locations with large migration angle for the FSS gradients of 0.1 and 0.2 Pa mm^−1^ (Figure 4(b), Supplementary Movies S2 to S5). When chemically blocking the pathways of MSCC, PLC, and ER and removing the extracellular calcium, the migration of cells toward the region with low FSS level was significantly reduced, but the migration along the flow direction was significantly enhanced compared with the control group (Figure 4(c,d)). Moreover, with the addition of inhibitors, cell migration angle decreased significantly (Figure 4(e)). These results suggested that migration of RAW264.7 cells toward the low-FSS region might be regulated by calcium signaling pathways.

## Discussion

The mineral homeostasis within the bone is mainly regulated by two coupled processes, namely, bone resorption and matrix formation [22]. Interestingly, some studies found that bone resorption usually occurs near the large-scale microcracks but do not diffuse damage [12–14]. The factors deciding the aggregation of osteoclast precursors targeting at the large-scale microcracks and final fusion into mature osteoclasts are still unclear [23–27]. One previous study adopted microfluidic technology to construct gradient FSS field and demonstrated that tumor cells prefer to adhere and grow at the low-FSS region, i.e., the inner side of curved microvessels [28]. To the best of our knowledge, however, the gradient FSS field for the parallel-plate flow system and its effect on cell migration have yet to be investigated. In the present study, we constructed a parallel-plate flow chamber with different gradients of FSS to investigate the influence of magnitude and gradient of FSS on the migration of osteoclast precursor RAW264.7 cells. One advantage of this modified parallel-plate flow chamber is to avoid the effect of secondary flow on cell migration compared with our previous study by using a cone-plate flow chamber. (The manuscript has been submitted to another journal. Some data of the cone-plate flow chamber have been provided in the supplementary Figure S8.)

In this work, RAW264.7 cells were exposed in the gradient FSS fields with the magnitude ranging from 0.1 Pa to 2.0 Pa, which were the physiological levels within the bone cavities [29]. When cells were applied under FSS for 2 h, they preferred to migrate toward the low-FSS region rather than along the flow direction. If extending the stimulation duration to 8 h under 0.1 Pa FSS, the results showed that there was no significant difference in migration speeds at different time points of 2, 4, 6, and 8 h, but the latter was significantly higher than the former at each time point (supplementary Figure S6), indicating that the cells can maintain their ability of migrating toward low-FSS region during 8 h-FSS stimulation. In high FSS gradient of 0.2 Pa mm^−1^ with low FSS of 0.1 Pa, the angle of cell migration is close to 90° (Figure 3(f)), almost perpendicular to the flow direction. When increasing the FSS magnitude for 0.2 Pa mm^−1^ FSS gradient, the cells tend to migrate along the flow direction. In low-FSS gradient of 0.05 Pa mm^−1^, the cells migrated toward the low-FSS region regardless of FSS magnitude (Figure 3(d)). When analyzing the cell migration for constant FSS level of 0.1 or 0.8 Pa, interestingly, the increasing FSS gradient enhances the cell migration toward the low-FSS region for 0.1 Pa but reduces it for 0.8 Pa (Figure 3 and supplementary Figure S7). These results imply that this FSS gradient-induced migration of osteoclast precursors occurs in the locations with low FSS level and high FSS gradient.

To elucidate the mechanism of FSS gradient-dependent migration of osteoclast precursors, we focused on the calcium signaling pathways related to mechanosensitive ion channel and ER. Two sources are available for the increase of [Ca [2]+]_i_, namely, the influx of extracellular calcium ions ([Ca^2+^]_0_) and the release of intracellular calcium stores. The extracellular calcium ions enter into a cell mainly through the plasma membrane ion channels including MSCC or voltage-sensitive calcium channels in osteoclasts. Previous studies demonstrated that extracellular calcium ions (Ca^2+^) regulate the localization and homing of osteoclasts or their precursors [30,31]. Our previous studies found that FSS can induce calcium response in either mature osteoclasts or the precursors and inhibiting MSCC or the ER pathway significantly reduced the flow-induced calcium response [21,32]. Our another study also demonstrated that steady fluid flow with uniform flow field drove the migration of RAW264.7 cells along flow direction, which was significantly reduced when blocking MSCC or ER pathways [15]. We assume that this interesting phenomenon of FSS gradient-dependent migration of RAW264.7 cells should be in connection with the opening of mechanosensitive ion channel under fluid flow. However, the exact molecular mechanism remains unknown.

Osteoclasts are derived from hematopoietic stem cells, and differentiate into mononuclear osteoclast precursors under the action of cytokines such as M-CSF [33]. Fatigue damage of bone may occur under long-term cyclic mechanical loading. Some studies indicate that there was a co-localization relationship between bone resorption and microcrack [25,34,35]. It was assumed that the recruitment of monocytes to the area of bone resorption and their fusion into multinucleated osteoclasts might be influenced by chemical or mechanical factors [36,37]. This osteoclast recruitment should be similar to metastatic homing of tumor cells from peripheral blood circulation to the bone. In addition, tumor cancer metastasis can lead to fracture, ostalgia, osteoporosis, etc. [38]. Therefore, the effect of mechanical stimulations especially the gradient FSS on the metastatic homing of tumor cells to the bone should be studied in the future.

In conclusion, a custom-made parallel-plate flow chamber was adopted in this study to construct flow field with gradient FSS. The results indicated that RAW264.7 cells migrate toward low-FSS region when FSS magnitude is small, but FSS gradient is relatively large. This FSS gradient-induced migration of RAW264.7 cells was regulated by calcium signaling pathways. This study may provide insights into the mechanism of the recruitment of osteoclast precursors in the region of bone resorption.
