# Supplementary figures and images for: Gradient fluid shear stress regulates migration of osteoclast precursors
Source: Cell Adh Migr. 2019 May 25;13(1):183–91. doi: 10.1080/19336918.2019.1619433 (PMC6550536; doi:10.1080/19336918.2019.1619433)

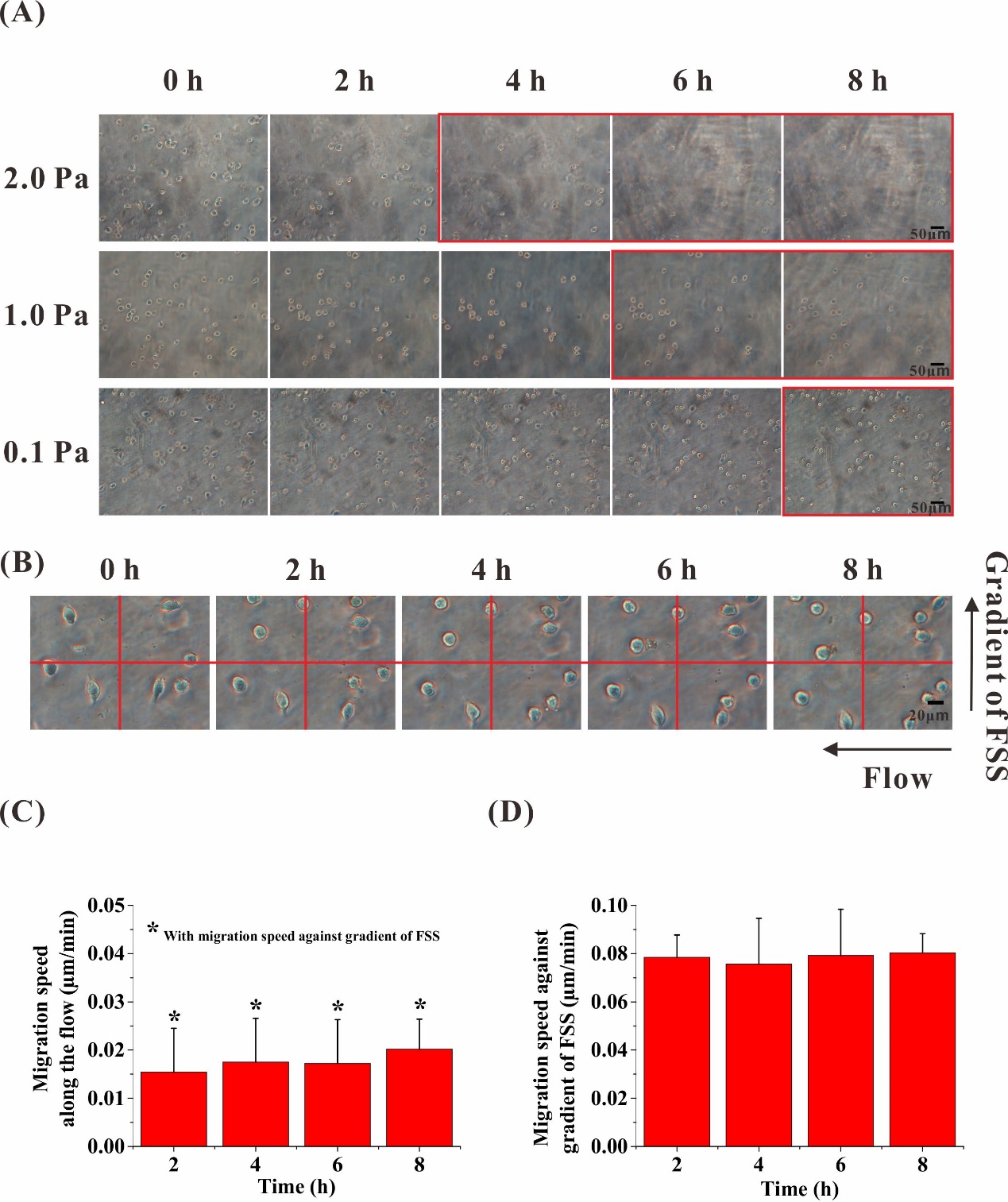

Supplement: Supplemental Material [file kcam-13-01-1619433-s001.zip › supplement S6.docx]

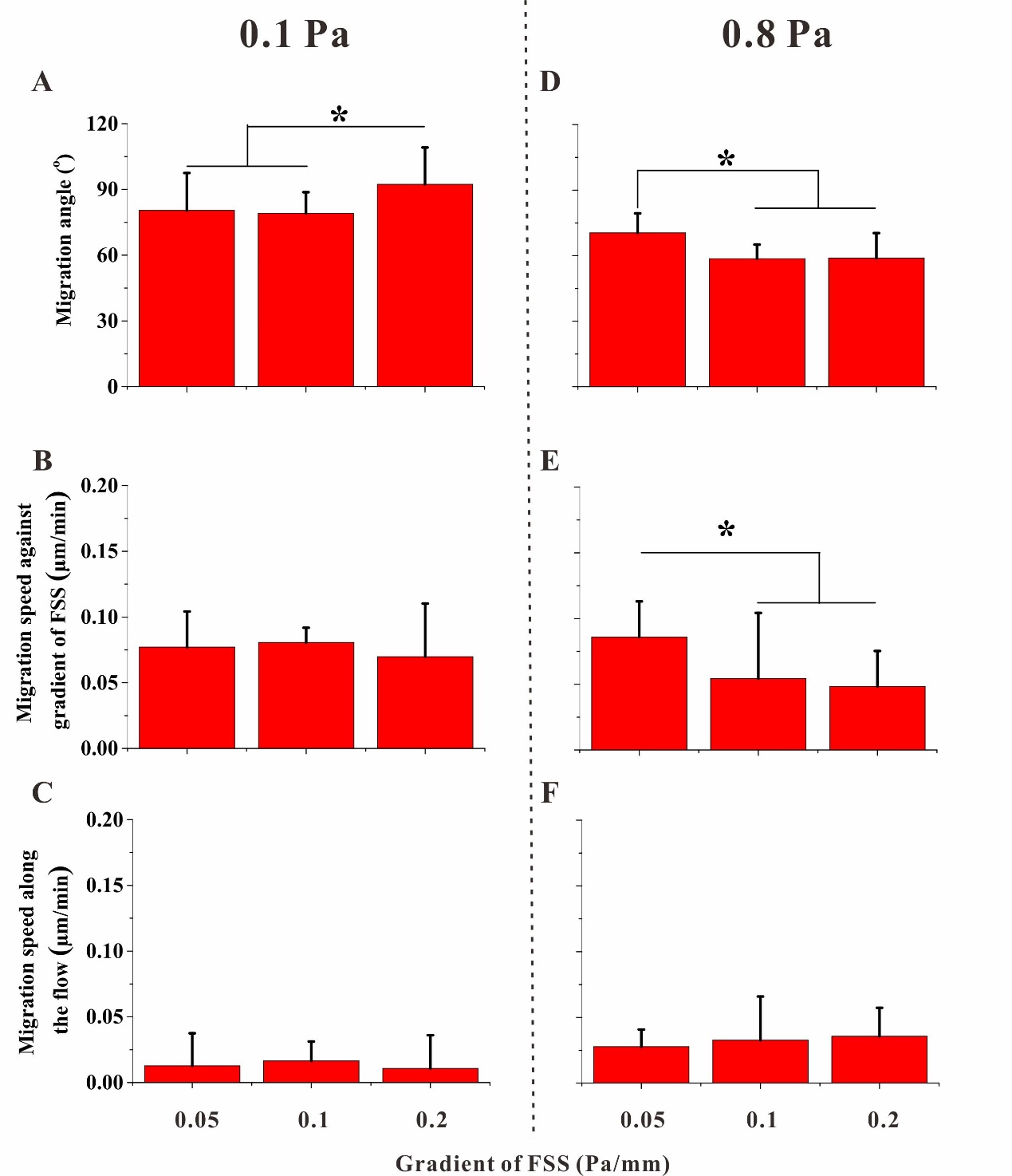

Supplement: Supplemental Material [file kcam-13-01-1619433-s001.zip › supplement S7.docx]

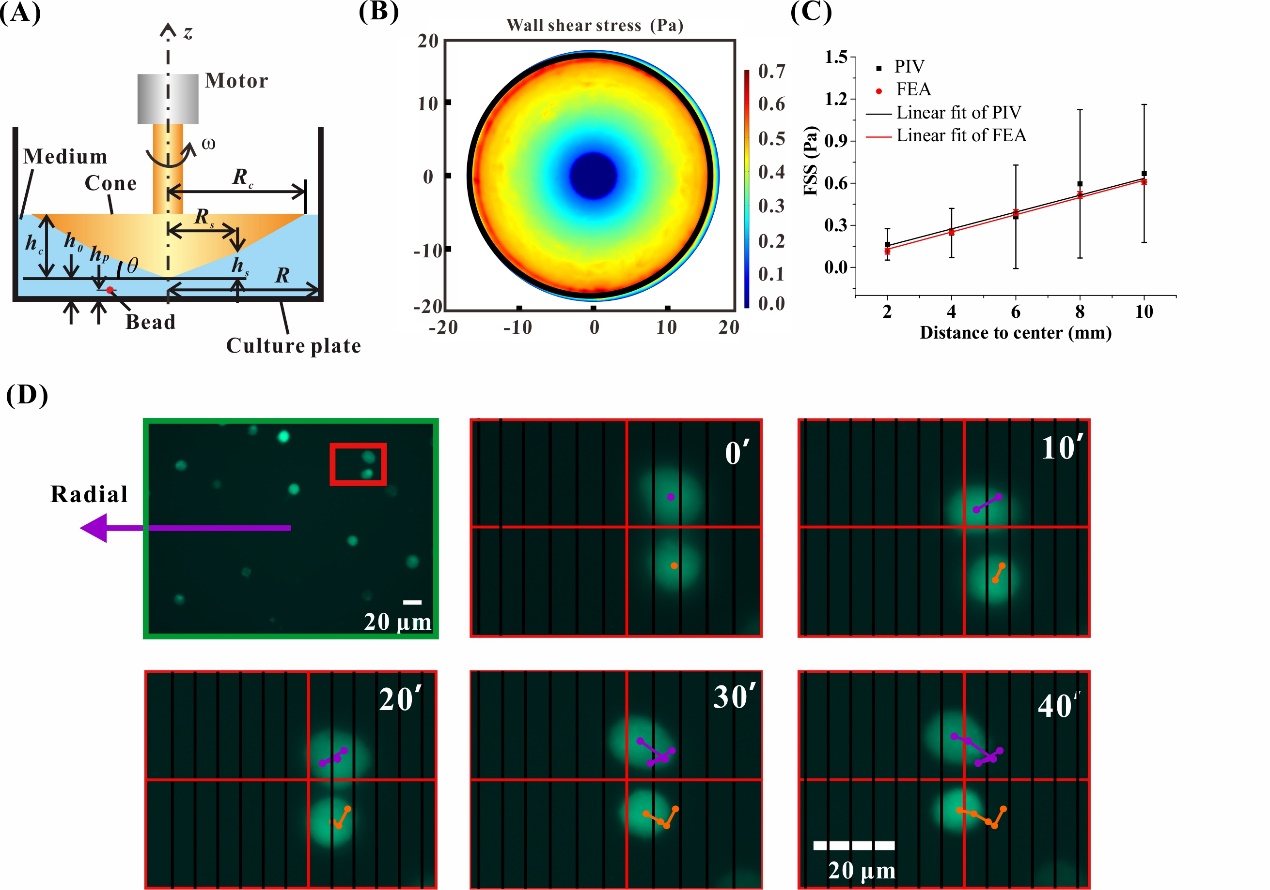

Supplement: Supplemental Material [file kcam-13-01-1619433-s001.zip › supplement S8.docx]
